# Impact of creative workshops in an institutionalized patient with moderate/severe cognitive impairment with behavioral disorders: A case report

**DOI:** 10.3389/fpsyt.2023.1132659

**Published:** 2023-03-09

**Authors:** Nuria Bravo-Garrido, Juan Francisco Morán-Cortés, Valle Coronado-Vázquez, María del Valle Ramírez-Durán

**Affiliations:** ^1^Department of Nursing, University of Extremadura, Plasencia, Spain; ^2^Department of Semiology and Clinical Communication, Universidad Francisco de Vitoria, Madrid, Spain

**Keywords:** intellectual disability, cognitive dysfunction, conduct disorder, creativity, pica, cooking, case report

## Abstract

The Hospital Care Unit for individuals with intellectual disabilities and behavioral disorders provides comprehensive care in a controlled and video-surveyed facility that minimizes access to potentially manipulative materials during aggression or pica episodes. The patient was admitted to the unit due to issues including ingestion of non-edible fluids, aggression toward staff and other patients, and self-injury. All patients participated in occupational activities led by an occupational therapist from Monday to Friday from 10 a.m. to 11:30 a.m. In addition, creative workshops such as cinema forums and cooking workshops were held on some afternoons. During the analyzed period from January to June 2022, the patient experienced three episodes of pica, 14 assaults toward staff, and eight toward peers. All of these incidents occurred after dinner and were triggered either by the inability to eat dessert or by refusal to brush teeth afterward. In our case study, the implementation of creative workshops such as cooking had a positive effect on decreasing instances of pica and aggression. These workshops slightly improved participation in other occupational therapy activities and stabilized the patient’s behavior, increasing the likelihood of her being able to return to her habitual residence.

## 1. Introduction

Pica is defined as the ingestion of non-edible substances including paper, small items of clothing, liquid detergent and grass. This behavior is common in persons with intellectual disabilities and can become life-threatening resulting in surgery, gastrointestinal obstructions, perforations, and death (1, 2).

Although there are some literature reviews about interventions to treat and cease pica, the success of the interventions and treatments are not definitive, mainly due to a lack of outcome studies (3–6). Furthermore, the majority of the literature refers to children or adults with autism or ADHD (7–10). Among the studied interventions, several emerge including self-protective devices, overcorrection, water mist and aromatic ammonia, behavioral interventions, brief restraint, differential reinforcement, discrimination training, patient self-report, and ecological modifications (5–7). Regarding brief restraint, they should be limited to extreme situations in which staff, other patients and the patient himself/herself need protection (11).

Along with pica, patients with intellectual disabilities often present other behavioral disorders that include aggressions. On this matter, patients might exhibit self-injury, physical or vocal aggression or destruction. Patients with moderate or severe intellectual disability and admitted to hospitals or living in community groups exhibit more aggressions (12, 13).

Aggressions carry burden and psychological distress for both aggressor and victim. For the aggressor, these events can alter their rehabilitation. For the victim and organization, the decrease in the efficacy and effectiveness of rehabilitative efforts, psychological distress including anxiety, anger and burnout, and physical injury (14, 15).

However, people with intellectual disabilities and behavioral disorders can be secured on a long-term basis from serious injury if appropriate management and interventions are both engaged (7). We present the case of a patient with moderate/severe intellectual disability whose pica and aggressions events decreased after introducing a cooking workshop.

## 2. Methods

A case study was analyzed and described.

### 2.1. Settings and subject

Created in 2013, the Hospital Care Unit for people with intellectual disabilities and behavioral disorders (UHDAC in Spanish) provides therapy to adults in a closed residence for a temporal period. This regional-referenced center offers integral care to a maximum of 14 people in a controlled and video-surveillance facility that minimizes access to susceptible material of being manipulated in either aggression or pica episodes.

The patient’s evolution and response to their intervention program determine the permanence in the unit, being the behavioral disorder treatment and progression determinant to the patient’s discharge.

We selected an admitted patient who, at the time of the analyzing period, had already spent 6 months in the unit being thus fully adapted. Furthermore, during the first 6 months and the following six (analyzed period) the patient never left the unit with the exception of medical appointments, being returned to it in the afternoon. The analyzed period comprised from January to June 2022. In this period, the care staff (nurses and nursing assistants) and admitted patients were constant, reducing any behavioral episode caused by changes in the staff, jealousy from the discharge of a peer or changing of settings (16).

### 2.2. Interventions

All patients participate in occupational activities led by an occupational therapist and created by her every Monday to Friday from 10 a.m. to 11:30 a.m. In these sessions patients perform activities that aim to develop and maintain social, emotional, cognitive and manipulative skills. Nonetheless, Friday activities tend to be more ludic and centered on self-care, rewarding attendance throughout the week at the other occupational activities. The occupational therapist counts with the support of monitors and nursing assistants supervising the patients uninterruptedly in a 2:1 ratio. These activities, while maintaining the same topic for everybody are individually adapted.

In addition, patients attended creative workshops on some afternoons. Specifically, they attended sessions of cinema-forum from January to March and cooking workshops from April to June. In order to participate in the creative workshops, it was necessary to comply with the rules of conduct established in the unit.

Cinema-forum took place in the Assembly Hall, which comprises 50 seats, and a large projection screen where personnel switch off the lights simulating being in a movie theater. Afterward, the users recreated the characters from their perspective by doing very low-demand role-playing. In addition, they discussed their feelings toward the characters, how they would label them and whether the movie brought any changes in their life. In this activity, they can develop their imagination, express feelings, and manage emotions.

The cooking workshops comprised easy-to-prepare dishes, mostly desserts, that were set by consensus among patients. They selected and prepared the ingredients with the help and guidelines from the therapist. The workshop concluded with a tasting as a reward and positive behavioral support for the task performed. The cooking workshop took place in a room equipped with all the utensils someone would find in a home kitchen: ceramic stove, oven, microwave, blender, utensils such as plates, cutlery, bowls, etc. Creativity was explicit through the ingredient modification to try new flavors, aromas, and textures. In addition, each one personalized the creation of their dish, showing it to the rest afterward.

We had access to all records from nurses, psychologists, occupational therapist and her medical history.

This study has been approved by the territorial management of the Service for the Promotion of Autonomy and Attention to Dependency (SEPAD in Spanish) in Cáceres (Extremadura) and informed consent has been given by the legal guardian of the patient.

### 2.3. Case description

The selected patient was a 48 years old female with congenital syphilis, moderate/severe intellectual disability and an IQ of 45 in the Wechsler scale. She was orphan without any family support.

The admission was due to severe behavioral alterations that made her management extremely difficult in her habitual residence despite pharmacological adjustment. The main alterations included non-eatable fluids ingestion, aggression toward staff and other residents, and self-injure facing frustration whenever her demands were not met. Pharmacological treatment was as follows: Risperidone depot one injection every 15 days, Carbamazepine (1-1-1), Trazodone (0-0-1), Fluoxetine (1-0-0), Valproic acid (1-0-2), Risperidone (3-2-3), Lormetazepam (0-0-1), and Clonazepam (1-1-1). At any patient admission, the psychiatrist revises the pharmacological treatment and modifies it when necessary. In this case, there was no treatment modification except for the introduction of rescue medication (Risperidone) when the patient showed behavioral crisis or disorders.

Table 1 shows the patient’s behavioral disorder events and consequences and interventions during the analyzed period. Behavioral records at the creative workshops are summarized in Table 2.

**TABLE 1:** Frequency of behavioral events, triggers, consequences, and interventions.

Month	No. of events	Probable cause/Trigger	Consequence/Interventions
January	One staff assault	Unable to eat dessert.	Mechanical restraint. Explanation of unit rules. Phase 0: no OT; stays in her bedroom.
February	Three staff assault	Agitation in the prior days needing chemical restraints.	Mechanical restraint. Explanation of unit rules. Phase 0: no OT; stays in her bedroom.
	One peer assault	Refusal to brush her teeth after consuming sugary desserts. Patient’s own birthday	Mechanical restraint. No desserts. Explanation of unit rules.
March	Five staff assault	Unable to eat dessert or the refusal to brush her teeth after eating it.	Mechanical restraint. Explanation of unit rules. Phase 0: no OT; stays in her bedroom.
	Four peer assault	Refusal to brush her teeth after consuming sugary desserts. Patient’s own birthday	Mechanical/chemical restraint. No desserts. Explanation of unit rules.
	One ingestion	Refusal to brush her teeth after consuming sugary desserts.	Mechanical restraint.
April	Four staff assault	Unable to eat dessert or the refusal to brush her teeth after eating it.	Mechanical restraint. Explanation of unit rules. Phase 0: no OT; stays in her bedroom.
	One peer assault	Refusal to brush her teeth after consuming sugary desserts. Patient’s own birthday	Mechanical restraint. No desserts. Explanation of unit rules.
	One ingestion	Refusal to brush her teeth after consuming sugary desserts and refusal to celebrate her birthday.	Mechanical restraint. Phase 0: no OT; stays in her bedroom.
May	One staff assault	Agitation and insomnia the night before.	Mechanical restraint. Explanation of unit rules. Phase 0: no OT; stays in her bedroom.
	One peer assault	Refusal to brush her teeth after consuming sugary desserts.	Mechanical restraint. No desserts. Explanation of unit rules.
	One ingestion	Refusal to brush her teeth after consuming sugary desserts.	Mechanical restraint. Phase 0: no OT; stays in her bedroom.
June	One peer assault	Refusal to brush her teeth after consuming sugary desserts.	Mechanical restraint. No desserts. Explanation of unit rules.

**TABLE 2:** Behavioral records at the creative workshops.

Cinema forum 1/12	Cinema forum 1/17	Cinema forum 2/14	Cinema forum 2/21	Cinema forum 28/2
Very participative, expressing interest in the film.	Aggression episode toward the audio visual material. She had to leave the session.	Due to misbehavior, she did not attend the session.	Cheerful and participative.	Due to misbehavior, she did not attend the session.
**Cinema forum 3/7**	**Cinema forum 3/30**	**Cooking workshop 4/28**	**Cooking workshop 5/5**	**Cooking workshop 5/23**
Nothing to report.	She fell asleep during the session.	She completed the activity (making a smoothie) and shared with peers when explained to do so. She was very happy to be able to choose the fruit she ate.	She completed the activity (making a chocolate-cookie pie). She enjoyed tasting the ingredients and being able to overstaffing the pie with chocolate. She wanted to eat the pie before it was finished.	Since she could not attend the cooking workshop (pastry making), she let the psychologist know that she wanted to leave the unit immediately. The occupational therapist invited her to a treat in the cafeteria and sorted it out.

During the analyzed period, the patient experienced three isolated episodes of pica in March, April, and May. Of these three episodes, the one in April was notable as it occurred on a weekday and was the only day of the week that the patient attended the occupational therapy program. The patient’s persistent refusal to brush her teeth after consuming sugary desserts was the trigger of all pica events. Furthermore, for the episode in April, the patient’s refusal to celebrate her birthday was another trigger. As for the aggressions that resulted in mechanical restraint, 14 assaults on the staff were recorded, with June being the only month without any recorded aggression. Additionally, all aggressions concentrated from February to April, totaling 12 assaults, all of which occurred in the evening.

Upon reviewing the assaults individually, the one in January took place in the evening. On that day, the patient’s physician had encouraged her to eat fewer sugary desserts and substitute them with healthier options implementing a reinforcement therapy in which the care staff would withdraw sugary desserts at lunch/dinner in the event of behavioral disturbance or non-compliance with unit rules. In February, the three cases of aggression occurred over the weekend, with the first and the last being preceded by days of agitation requiring pharmacological intervention. The manipulation of plugs and the intervention of the care staff to prevent ingestion mediated all five instances of aggression that occurred in March. These incidents all took place after dinner and the triggers were either the inability to eat dessert or the refusal to brush teeth after eating dessert. The nursing record shows that the patient becomes agitated around the approach of her birthday in April and the potential for purchasing desserts to celebrate it.

The four instances of aggression recorded in April occurred on the weekends of April 8th and 12th. All of these incidents occurred after dinner and the triggers were the same, either a lack of ability to eat dessert or a refusal to brush teeth after eating it. The aggression toward the new occupational therapist in May was preceded by insomnia at night and agitation due to the desire to host a party.

**FIGURE 1:**
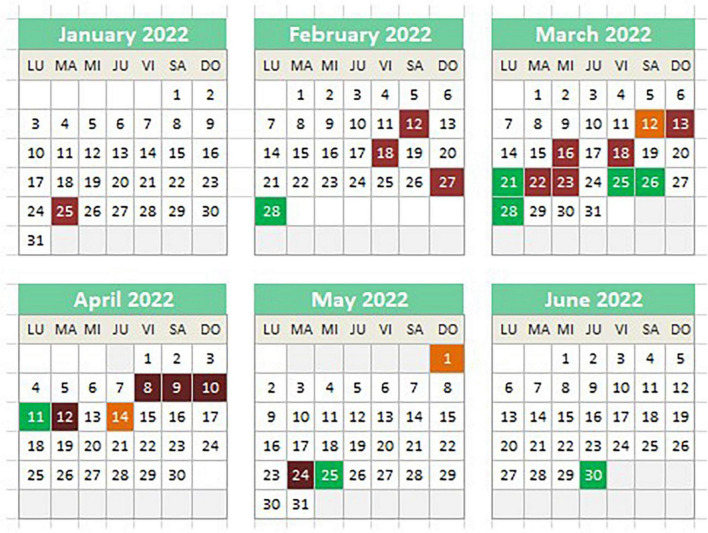
Calendar showing the registered alterations. Red, assault on staff; Green, assault on peers; Orange, ingestion.

Regarding the incidents of peer assault, eight resulted in the use of mechanical or chemical restraints. Four of these incidents occurred in the week of March 21st to 28th. These assaults on peers were characterized by hair-pulling and self-harm in the form of head-butting, typically occurring in the evening and triggered by the anticipation and excitement of celebrating their birthday and being able to go out and purchase desserts. The other incidents of aggression, which occurred in February, April, May, and June, displayed a similar pattern: hair-pulling triggered by the individual’s birthday or refusal to brush her teeth after consuming dessert. The calendar with these events is depicted in Figure 1.

## 3. Discussion

Since the implementation of cooking workshops, there have been no reported events of behavioral changes related to ingestion, except for one when the patient was unable to eat the food prepared in the workshop. Aggressive episodes requiring mechanical or chemical restraints have also decreased from nineteen events of staff or peer assault prior to the beginning of the cooking workshops to three isolated events in the subsequent period.

Upon reviewing the literature, we discovered several interventions for reducing and eliminating pica (5–7), including replacing a potential item for consumption with a preferred food choice of the patient. Carter (17) reports the disappearance of pica in a 72 years-old woman after 2 years of using chocolate, biscuits, coffee, and soft drinks as substitutes. In another case study involving two children with developmental disabilities (18), they were taught to replace a particular pica item with a preferred snack food, starting with a 1:1 ratio and gradually decreasing to a 3:1 ratio. While this intervention may be effective for the patient in question, the fact that access to sugary food and refusal to follow rules concerning such food (e.g., brushing teeth after dessert) was a trigger for behavioral changes may lead to the patient purposely inducing pica episodes in order to obtain access to sugary treats.

The fact that a reinforcement therapy withdrawing sugary foods when either behavioral events were present or the patient did not comply with the unit rules should be noted as a factor in the behavioral disorders. We hypothesize that participation in cooking workshops where desserts are available can provide the patient with a sense of peace and the assurance of double access if she adheres to the rules.

According to Ashworth et al. (1), episodes of pica in individuals with intellectual disabilities are often correlated with a lack of family support, social contact, lack of participation in activities of interest or a daily schedule, and lack of involvement in recreational activities. In our case, the patient does not engage in Occupational Therapy activities due to a lack of compliance with rules of coexistence or lack of interest, resulting in missed activities for up to 7 days in a row. When comparing the patient’s attitudes toward cinema-forum workshops with those toward cooking workshops, there is a noticeable change in attitudes and how the patient expresses a desire to attend the cooking workshops and therefore, the regular occupational therapy activities (mandatory in order to attend the afternoon creative workshops).

Regarding aggressions, there is a significant association between behavior disorders and the severity of the intellectual disability, living in restrictive settings and coercive measures (restraints) (12, 15). In our case, this fact poses a dilemma. The reason for admitting her to the unit was the presence of behavioral disorders and the impossibility of maintaining cohabitation in her habitual home.

A known trigger for aggression among intellectual disability people is feelings of frustration (10, 16). In our case, it was clear that the main trigger was feelings of frustration whenever her demands related to food were unmet. On this matter, the literature suggests that meeting a person’s needs better would likely result in fewer events of behavioral disruptions (13), thus, her cooking workshop attendance might reveal another path to meet her needs. However, more research regarding aggression and its prevention is needed.

AThis study has some limitations, including the settings, pharmacological treatment, care staff supervision and health staff records which sometimes were scarce. Furthermore, the potential influence of the patient’s birthday occurring during the analyzed period and the limited sessions of the cooking workshops constrained the possibility of demonstrating long-term efficacy. Implementing a longer-planned intervention controlling confounding variables could offer more reliable data. Notwithstanding, our patient experienced a decrease in episodes of pica and aggression, making progress on their path to recovery and potentially returning to their habitual residence in the future.

## 4. Conclusion

In our case study, the incorporation of cooking workshops, had a positive impact on reducing occurrences of pica and aggression toward staff in the analyzed intellectually disabled patient who was institutionalized. These workshops also slightly improved participation in other occupational therapy activities and stabilized the patient’s behavior, increasing the likelihood of her being able to return to her usual residence.

## Data availability statement

The original contributions presented in this study are included in the article/supplementary material, further inquiries can be directed to the corresponding author.

## Ethics statement

The studies involving human participants were reviewed and approved by Territorial Management of the Service for the Promotion of Autonomy and Attention to Dependency. The patients/participants provided their written informed consent to participate in this study. Written informed consent was obtained from the individual(s) for the publication of any potentially identifiable images or data included in this article.

## Author contributions

NB-G and MR-D conceived and carried out the study, collected and analyzed the data, and draft the manuscript. JM-C and VC-V helped draft the manuscript. All authors read and approved the final manuscript.
